# Non‐oral, aerobic, Gram‐negative bacilli in the oral cavity of Thai HIV‐positive patients on Highly‐active anti‐retrovirus therapy medication

**DOI:** 10.1111/jicd.12387

**Published:** 2019-01-30

**Authors:** Pratanporn Arirachakaran, Sureeat Luangworakhun, Georgios Charalampakis, Gunnar Dahlén

**Affiliations:** ^1^ Department of Oral Medicine Faculty of Dentistry Chulalongkorn University and Dental Center Bangkok Hospital Bangkok Thailand; ^2^ Oral Research Center Faculty of Dentistry Chulalongkorn University Chulalongkorn Thailand; ^3^ Department of Oral Microbiology and Immunology Institute of Odontology Sahlgrenska Academy University of Gothenburg Gothenburg Sweden

**Keywords:** aerobic Gram‐negative bacilli, antibiotic susceptibility, extended spectrum beta‐lactamase, HAART medication, HIV positive

## Abstract

In the present study, we identified and evaluated the antibiotic susceptibility of 96 independent, aerobic, Gram‐negative bacillus isolates from 255 Thai HIV‐positive adults who were on Highly‐active anti‐retrovirus therapy (HAART) medication. Another 46 isolates from HIV non‐HAART individuals, vertically transmitted HIV‐positive individuals, and non‐HIV controls were included for comparison. A total of 103 strains were tested for antibiotic susceptibility using disc diffusion for screening and E‐test for minimal inhibitory concentration determination, with special attention on extended‐spectrum beta‐lactamase (ESBL) isolates. *Pseudomonas aeruginosa*,* Pseudomonas luteola*,* Burkholderia cepacia*,* Aeromonas hydrophila*,* Klebsiella*, and *Enterobacter* species were the most common bacteria. All strains were resistant against penicillin, amoxicillin, clindamycin, and metronidazole. No ESBL isolates were found.

## 1 INTRODUCTION

Oral microbiota, although its complexity, is normally in a balanced condition with the host termed “microbial homeostasis.”^1^ This balance can be destroyed by a number of factors.^2^, ^3^ Systemic diseases, Medication, and immune‐compromised conditions are well‐known general factors that could lead to overgrowth by non‐oral microorganisms (dysbiosis), and opportunistic infections might follow.^4^ Overgrowth with yeasts is most frequent, but bacteria, such as *Staphylococcus aureus*, enterococci, and aerobic, Gram‐negative bacilli (AGNB) also frequently occur in opportunistic oral infections.^5^ These microorganisms could be present in the transient microbiota of the oral cavity, but are not considered oral commensals; they are considered non‐oral “exogens.”^6^

The impact of AGNB when they occur in the oral cavity is unclear and controversial. They are commonly associated with oral mucosal infections in immune‐compromised and hospitalized patients.^4^, ^5^ In these patients, oral mucosal infections might spread to the respiratory system and cause life‐threatening infections.^7^, ^8^ One concern is that these bacteria are seldom identified at the species level, but are referred to as “enterics” and “pseudomonads.”^5^, ^9^, ^10^, ^11^ Species and genera within the group of AGNB might have similarities, but the group consists of a wide spectrum of bacterial species, which are disparate in virulence, pathogenicity, and antibiotic susceptibility. A closer specification of AGNB is needed, as they are frequently are multiresistant to common antibiotics used in dentistry. A major concern is the presence of the extended‐spectrum beta‐lactamase (ESBL) resistant isolates, which to the best of our knowledge, have not been isolated from the human oral cavity. In a previous study on the presence of opportunistic microorganisms in HIV‐positive individuals, the frequency and load of such bacteria in the Highly‐active anti‐retrovirus therapy (HAART) group were similar to those of the non‐HAART group, but significantly higher than those of HIV‐negative controls.^11^ In the present study, we report on the species identification and antibiotic susceptibility of AGNB isolates from the oral cavity of HIV‐positive individuals on HAART medication, with special attention to the presence of ESBL isolates.

## 2 MATERIALS AND METHODS

### 2.1 Bacterial strains

A total of 142 AGNB strains were used in the present study. They were originally isolated from patients attending the Infectious Diseases Clinic at the Faculty of Dentistry, Chulalongkorn University, Bangkok, Thailand.^11^ Of 255 Thai HIV‐positive adults on HAART medication, 96 independent AGNB strains were isolated from 87 AGNB‐positive individuals; nine individuals had two different species. Another 46 species were isolated from 20 HIV non‐HAART individuals (8 strains), 19 from 53 vertically‐transmitted HIV‐positive individuals, and 19 from 30 non‐HIV controls, which were included for comparison. Sixteen individuals had two different species. The strains were originally isolated in samples taken from dorsum of the tongue, gingiva, and if present, a deep periodontal pocket or a mucosal lesion, as previously described elsewhere.^11^ If the same species was present in more than one sample, the isolate from the tongue was chosen for the study. Thus, 127 strains were from the tongue and 16 from the gingiva.

The cultivation analysis followed the methods, as described in elsewhere.^11^ A McConkey agar plate was incubated for 2‐3 days, specifically for the enumeration and differentiation of AGNB. Each isolate was checked for colony morphology and cell morphology through Gram staining. The ability to grow on McConkey agar by lactose and non‐lactose fermentation was recorded. Tests for oxidase and catalase, and the ability to reduce nitrate were performed according to Lennette et al.^12^ Identification was further performed using commercially systems. API RapiD20E (Biomerieux, St Louis, MO, USA) was used to identify *Enterobacteriaceae* in 4 hours, while API 20 NE (Biomerieux) was used for lactose‐negative isolates. Bacterial suspensions were prepared in 0.85% NaCl for inoculation into the eight conventional substrates and in AUX medium (Biomerieux) for inoculation into the 12 assimilation cupules. The tests were incubated for 24 hours at 30°C, and the test results were read in a computer program (APIweb; Biomeriaux). The test was repeated for strains when discrimination was <90%. Reference strains of AGNB were used as positive controls in the identification procedures: *Escherichia coli* ATCC 25922 (American Type Culture Collection), *Klebsiella oxytoca* OMGS1762 (Department collection, Oral Microbiology, Gothenburg, Sweden) and *Pseudomonas aeruginosa* OMGS 1854. All isolates were stored at −80°C.

### 2.2 Antibiotic susceptibility

Routine screening for antibiotic susceptibility was performed using blood agar plates and the disc diffusion method (Oxoid, Basingstoke, UK) against six antibiotics commonly used in dentistry: penicillin V, isoxapenicillin, amoxicillin, clindamycin, erythromycin, and tetracycline. After incubation, the diameter of the inhibition zone of each strain was measured, and the strains were graded as sensitive, intermediate, and resistant. Minimal inhibitory concentration (MIC) was determined using the E‐test method (Biomerieux) against doxycycline, gentamicin, ciprofloxacin, norfloxacin, ceftibuten, cefotaxime, and ceftazidime. The MIC were read from the intercept where the ellipse inhibition zone intersected with the scale. European Committee on Antimicrobial Testing recommendations cut of levels for resistance were used.^13^

Screening and confirmatory test for ESBL production, and discs with ceftazidime (30 μg) and cefotaxime (30 g) were placed on the media with the test inoculum and incubated for 24 hours. Bacterial isolates showing ≤22 mm ceftazidime (corresponding to 2 μg/mL) and ≤27 mm cefotaxime (corresponding to 2 μg/mL) zones were suspected to be ESBL producers according to National Committee for Clinical Laboratory Standards and Center for Disease Control and Prevention.^14^ For ESBL confirmation, a combined disc method was used using ceftazidime and cefotaxime with clavulanic acid discs (30 μg; Oxoid, UK).

## 3 RESULTS

The identification of AGNB species in the oral cavity (predominantly from the dorsum of the tongue) of the 142 strains isolated as independent strains from 117 participants is shown in Table 1. The most frequent species found in the HIV‐positive individuals on HAART medication were lactose non‐fermenting, but oxidase‐positive, strains of *Pseudomonas* spp 24 (predominantly *Pseudomonas aeruginosa* and *Pseudomonas luteola*) and *Burkholderia cepacia* complex 6, *Aeromonas* spp 10 (*Aeromonas hydrophila*), while the lactose‐fermenting and oxidase‐negative *Enterobacteriaceae* belonging to *Enterobacter* spp 20 (predominantly *Enterobacter aerogenes*,* Enterobacter sakazakii* [now *Cronobacter sakazakii*]) were *Klebsiella* spp 14 (predominantly *Klebsiella oxytoca* and *Klebsiella pneumonia*), *Raoultella ornithinolytica* 5, *Stenotrophomonas maltophilia* 5, and *Serratia* spp 8 (*Serratia odorferia*,* Serratia ficaria*, and *Serratia liquefaciens*), while only one “coliform rod” identified as *Escherichia coli* was found. One strain of *Morganella morganii*, one strain of *Acinetobacter baumannii* (now *Acinetobacter wolfii*), one strain of *Delftia acidovorans*, and two strains of *Vibrio haemolyticus* were also found. Three strains could not be identified. There was no significantly different pattern in the distribution of the species or genera found among the AGNB isolates from other HIV groups and the non‐HIV controls (Table 1).

**Table 1:** Identification and number of aerobic, Gram‐negative bacillus isolates from the oral cavity of Thai HIV‐positive individuals compared with non‐HIV controls

	Total (N = 358)	HAART (N = 255)	HIV non‐HAART (N = 20)	HIV vertical (N = 53)	Non‐HIV (N = 30)
*Pseudomonas aeruginosa*	16	13	2	1	0
*Pseudomonas luteola*	17	10	1	5	1
*Pseudomonas putida*	1	0	1	0	0
*Pseudomonas stutzeri*	1	1	0	0	0
*Stenotrophomonas maltophilia*	5	4	0	0	1
*Burkholderia cepacia*	12	6	1	2	3
*Burkholderia pseudomallei*	1	1	0	0	0
*Burkholderia gladioli*	1				1
*Aeromonas hydrophila*	13	10	0	1	2
*Aeromonas aerogenes*	1	0	0	1	0
*Klebsiella pneumoniae*	12	8	0	2	2
*Klebsiella oxytoca*	12	6	0	1	5
*Raoultella ornithinolytica*	9	5	1	1	2
*Enterobacter aerogenes*	8	6	0	1	2
*Enterobacter gergoviae*	2	2	0	0	0
*Enterobacter cloacae*	3	3	0	0	0
*Cronobacter sakazaki (Enterobacter sakazaki)*	6	6	0	0	0
*Pantoea agglomerans (Enterobacter agglomerans)*	4	3	0	1	0
*Serratia odorferia*	4	3	1	0	0
*Serratia ficaria*	4	3	1	0	0
*Serratia liquefaciens*	2	2			
*Escherichia coli*	1	1	0	0	0
*Acinetobacter baumannii*	1	1	0	0	0
*Morganella morganii*	1	1	0	0	0
*Delphia acidovorans*	1	0	0	1	0
Unidentified NLF+oxidase (negative)	1	0		1	
Unidentified	2	1		1	
Total isolates (no. participants)	142 (117)	96 (87)	8 (5)	19 (14)	19 (11)

NLF, non‐lactose fermenting.

All 142 strains were shown to be resistant against penicillin V, amoxicillin, clindamycin, and metronidazole using the disc diffusion test (data not shown). MIC estimation using E‐test of 103 of the isolated AGNB is shown in Table 2. Ciprofloxacin showed activity against most strains, except for *Stenotrophomonas* spp, *Burkholderia cepacia* complex, and *Aeromonas* spp, which showed resistance in 80%, 11.1%, and 30% of the strains, respectively. A similar resistance pattern was shown for norfloxacin, ceftibuten, cefotaxime, and ceftazidime. No ESBL strains were found.

**Table 2:** Antibiotic susceptibility (% resistant strains) of 103 isolated aerobic, Gram‐negative bacillus strains grouped according to genus level

Strain (genus)	N	Doxycycline	Gentamicin	Ciprofloxacin	Norfloxacin	Ceftibuten	Cefotaxime	Ceftazidime	Ceftibuten + Ceftazidime + CA^a^
*Pseudomonas* spp	29	96.6	20.7	0	3.4	24.1	20.7	3.4	0
*Stenotrophomonas* spp	5	100.0	80	80.0	60.0	80.0	60.0	40.0	0
*Burkholderia* spp	9	89.0	33.3	11.1	12.5	37.5	44.4	37.5	0
*Aeromonas* spp	11	91.0	54.5	30.0	45.5	80.0	50.0	0	0
*Klebsiella* spp	20	100.0	5.0	0	5.0	9.1	0	9.1	0
R*aoultella* spp	5	100.0	0	0	0	0	0	0	0
*Enterobacter* spp	13	100.0	0	0	15.3	7.7	0	0	0
*Serratia* spp	5	0	0	0	0	0	0	0	0
*Vibrio haemolyticus*	2	100.0	50.0	100.0	100.0	50.0	50.0	0	0
Other^a^	4	50.0	0	0	0	0	0	0	0

Combination of ceftibuten, cefotaxime, and clavulanic acid for the identification of extended‐spectrum beta‐lactamase isolates.

a *Escherichia coli*,* Morganella morganii*,* Acinetobacter baumannii*,* Delftia acidovorans*.

## 4 DISCUSSION

The most common AGNB species isolated in the present study were lactose non‐fermenting, but oxidase‐positive, strains classified as *Pseudomonas* spp (*Pseudomonas aeruginosa* and *Pseudomonas luteola*), *Burkholderia cepacia* complex, and *Aeromonas* spp (*Aeromonas hydrophila*), which are normally found in water, food, and plants, but occasionally cause nosocomial infections in immune‐compromised and hospitalized patients.^7^ *Pseudomonas* spp can occur in the transient oral microbiota, but rarely colonize the oral cavity, which is due to their strong aerobic character. Later studies using molecular biology methods have shown that their presence might be underestimated,^15^ and their presence in the oral cavity has been found to be higher in complex biofilms, such as dental plaque. *Pseudomonas* spp are considered to be the main pathogen in chronic obstructive pulmonary disease and in biofilms on vehicles at intubation,^16^, ^17^ and consequently, their occurrence in the oral cavity should be recognized in immune‐compromised and/or hospitalized patients for the risk of spreading to the respiratory tract.

The remaining AGNB isolates were predominantly lactose‐fermenting and oxidase‐negative *Enterobacteriaceae*, commonly referred to as “enterics,” “enteric rods,” or “coliform rods.” They are predominantly classified into the genera of *Enterobacter* (*Enterobacter aerogenes* and *Enterobacter sakazakii* [now *Cronobacter sakazakii*]), *Klebsiella* (*Klebsiella oxytoca* and *Klebsiella pneumonia*), *Raoultella* (*Raoultella ornithinolytica*), *Stenotrophomonas* (*Stenotrophomonas maltophilia*), and *Serratia* (*Serratia odorferia*,* Serratia ficaria*, and *Serratia liquefaciens*). Only one coliform rod, identified as *Escherichia coli* was isolated, which was somewhat surprising. To avoid misunderstandings, the designation “coliform rods” should not be used in the context of oral microbiota, because they are more likely to be something else than *Escherichia coli*. *Enterobacter* spp and *Klebsiella* spp are commonly related to hospital infections and affect immune‐compromised patients with oral mucosal infections.^4^, ^5^ Taken together, none of these AGNB were more specifically associated to any of the HIV or non‐HIV groups. They were generally present in low numbers,^11^ which might indicate that their prevalence is in fact higher, but could be missed in the sampling process. Even considering presence in higher numbers (moderate/heavy loads corresponding to >10^3^ colony‐forming units), indicating a more colonized state of the bacteria, they did not differ between the HIV or non‐HIV groups.^11^ The majority of the samples (72.7%) from the gingiva also showed the same isolates from the dorsum of the tongue, indicating that AGNB might colonize mucosal surfaces in general, and are not necessarily associated to gingiva and periodontal disease specifically.^12^

The increase of AGNB after antibiotic therapy could be explained by the multiresistance of AGNB.^18^ The present study confirmed almost 100% resistance against antibiotics commonly used for oral infections and in periodontal therapy (penicillin, amoxicillin, clindamycin, and metronidazole). Ciprofloxacin and norfloxacin seem to be the drugs of choice once there are infections with AGNB that need antibiotic treatment. However, it is strongly recommended that antibiotics not be used for the treatment of surface infections/colonization even in immune‐compromised patients, such as those who are HIV positive or immune‐compromised, unless there is a risk of mortality. No ESBL strains were found in these Thai patients; however, this type of multiresistance has been reported to be frequent (>50%) in stool samples in Thailand.^18^ This could be due to the fact that *Escherichia coli* was rarely isolated in oral samples in the present study, but were the most common ESBL isolate in the stool samples. Some bacteria, such as *Stenotrophomonas maltophila*, are known to have multiresistance, which was also confirmed in the present study.

Non‐oral AGNB that are isolated from the oral cavity of Thai HIV‐positive individuals on HAART medication showed great genus and species diversity, and were highly resistant to common antibiotics.
